# Predicting dynamic changes of Ki-67 in breast cancer after neoadjuvant therapy based on multi−phase DCE-MRI delta-radiomics

**DOI:** 10.3389/fonc.2026.1793905

**Published:** 2026-04-20

**Authors:** Xuan Zhang, Haifeng Zhao, Hao Zhang

**Affiliations:** 1 The First Clinical Medical College of Lanzhou University, Lanzhou, China; 2 Department of Radiology, the First Hospital of Lanzhou University, Lanzhou, China; 3 Affiliated Hospital of Gansu University of Chinese Medicine, Lanzhou, China

**Keywords:** breast cancer, delta-radiomics, multi−phase DCE-MRI, neoadjuvant therapy, nomogram

## Abstract

**Background:**

Ki-67 is a key biomarker of tumor proliferation in breast cancer. A reduction in Ki-67 following neoadjuvant therapy (NAT) reflects chemosensitivity and holds significant prognostic value. Therefore, pre-treatment assessment of Ki-67 dynamics during NAT is crucial for evaluating patient prognosis.This study aims to predict the change in Ki-67 index in breast cancer patients following NAT using radiomic features derived from DCE-MRI.

**Methods:**

This retrospective study enrolled 148 breast cancer patients who underwent surgical resection after 6–8 cycles of NAT, randomly divided into training (n=104) and test cohorts (n=44) at a 7:3 ratio. Multivariable logistic regression (P<0.05) identified independent clinical risk factors for Ki-67 downgrading. Radiomics features were extracted from pre-treatment DCE-MRI scans from the early, peak, and delayed phases, along with the corresponding phase differences (delayed–early, delayed–peak, peak–early). Feature selection was performed with Principal Component Analysis (PCA) followed by Recursive Feature Elimination (RFE), and radiomics and delta-radiomics models were built using the LR-Lasso algorithm.The DeLong test compared AUC values to identify the optimal model. Top radiomics features were then combined with clinical factors to construct a hybrid model, evaluated by AUC, calibration, and decision curve analysis (DCA).

**Results:**

ROC curve analysis demonstrated that the peak-to-early delta-radiomics model achieved the best diagnostic performance in the testing cohort with an AUC of 0.817(95% CI: 0.685–0.949), significantly outperforming the delayed-to-early delta model [AUC = 0.648(95% CI: 0.484–0.812)]and the standalone peak-phase model [AUC = 0.615(95% CI: 0.444–0.785)]. Logistic regression analysis revealed that HER2 status (p = 0.031) and histological grade (p<0.001) were significant predictors for constructing the clinical-radiological model. Then, integrating the optimal delta-radiomics model with independent clinical-radiological risk factors to form a combined model increased the AUC to 0.851(95% CI: 0.740-0.960), which was significantly superior to both the clinical-radiological model alone [AUC = 0.785(95% CI:0.649-0.922)]and the delta-radiomics model alone. In the external validation cohort, this model [AUC = 0.919(95% CI:0.846-0.992)] also demonstrated superior performance compared to either the standalone clinical-radiological model [AUC = 0.801(95% CI:0.680-0.924)] or the delta-radiomics model [AUC = 0.803(95% CI:0.678-0.928)].

**Conclusions:**

Delta-radiomics based on MRI, combined with clinical parameters, represents a promising non-invasive approach for more accurately predicting Ki-67 downstaging in breast cancer following NAT, outperforming conventional radiomics models. Integrating radiomic features with clinical information holds the potential to further optimize individualized treatment strategies and improve prognostic assessment for breast cancer patients.

## 1 Introduction

Breast cancer(BC)is the most common malignancy and leading cause of cancer death in women worldwide (1). In recent years, neoadjuvant therapy (NAT) has gained broad clinical recognition in systemic breast cancer management, initially for high-risk or advanced cases, now also to increase eligibility for breast-conserving surgery (2, 3). During NAT, monitoring treatment response is crucial to guide next steps—surgery if pathologic complete response(pCR)is achieved, or stopping therapy if disease progresses (4, 5). Consequently, pretreatment prediction of response is key to minimizing complications and optimizing decisions.

Prognostic factors for most breast cancers include tumor size, histological type, grade, axillary lymph node metastasis, peritumoral lymphatic/vascular invasion, and expression status of estrogen receptor (ER), progesterone receptor (PR), human epidermal growth factor receptor 2 (HER2), and Ki-67 (6). Ki-67 is an important marker for assessing treatment response in breast cancer, and the accuracy of its detection results is crucial for patient prognosis evaluation and treatment strategy formulation (7). High Ki-67 expression is typically associated with increased risk of recurrence and reduced survival rates, and it has predictive value for pathological complete response (pCR) (8). Following neoadjuvant therapy, Ki-67 expression levels are significantly decreased compared to pre-treatment levels, serving as an effective indicator of chemotherapy sensitivity (9). Its dynamic changes hold clear prognostic significance: a decrease often suggests better survival outcomes, whereas an increase indicates elevated recurrence risk and poorer prognosis (10). Therefore, the dynamic changes in Ki-67 are considered a new potential prognostic marker. Compared to static Ki-67, these changes during treatment provide more precise prognostic information and are expected to serve as an effective tool for risk stratification following neoadjuvant therapy, offering key guidance for subsequent adjuvant treatment strategies (11). However, despite its recognized clinical value, standardizing Ki-67 assessment remains challenging due to methodological variability and poor reproducibility across observers and labs (12). Thus, a noninvasive approach for prediction is urgently needed.

Dynamic contrast-enhanced magnetic resonance imaging (DCE-MRI) is an established clinical modality for breast cancer evaluation (13). DCE-MRI captures tissue perfusion and contrast kinetics via sequential pre- and post-contrast imaging, reflecting tumor angiogenesis. Radiomics decodes tumor heterogeneity by extracting and analyzing quantitative imaging features to assess aggressiveness, grading, and prognosis (14). However, conventional radiomics often overlooks hemodynamic temporal dynamics. Delta-radiomics addresses this issue by continuously monitoring changes in imaging over time, thereby enabling precise and dynamic characterization of tumor biological evolution. And its analytical time window typically covers pre- and post-treatment periods and can also be extended to multiphase dynamic scans (15, 16). Delta-radiomics features are defined as the net change obtained by directly subtracting features from two different phases (17).While existing studies predominantly focus on baseline Ki-67 prediction using DCE-MRI radiomics, limited predictive models exist for NAT induced Ki-67 changes. Therefore, this study was designed to develop a novel delta radiomics model based on multi-phase DCE-MRI to quantitatively predict the change in Ki-67 expression following NAT in breast cancer patients, thereby providing a noninvasive tool for monitoring treatment response.

## 2 Materials and methods

### 2.1 Patients

**Figure 1:**
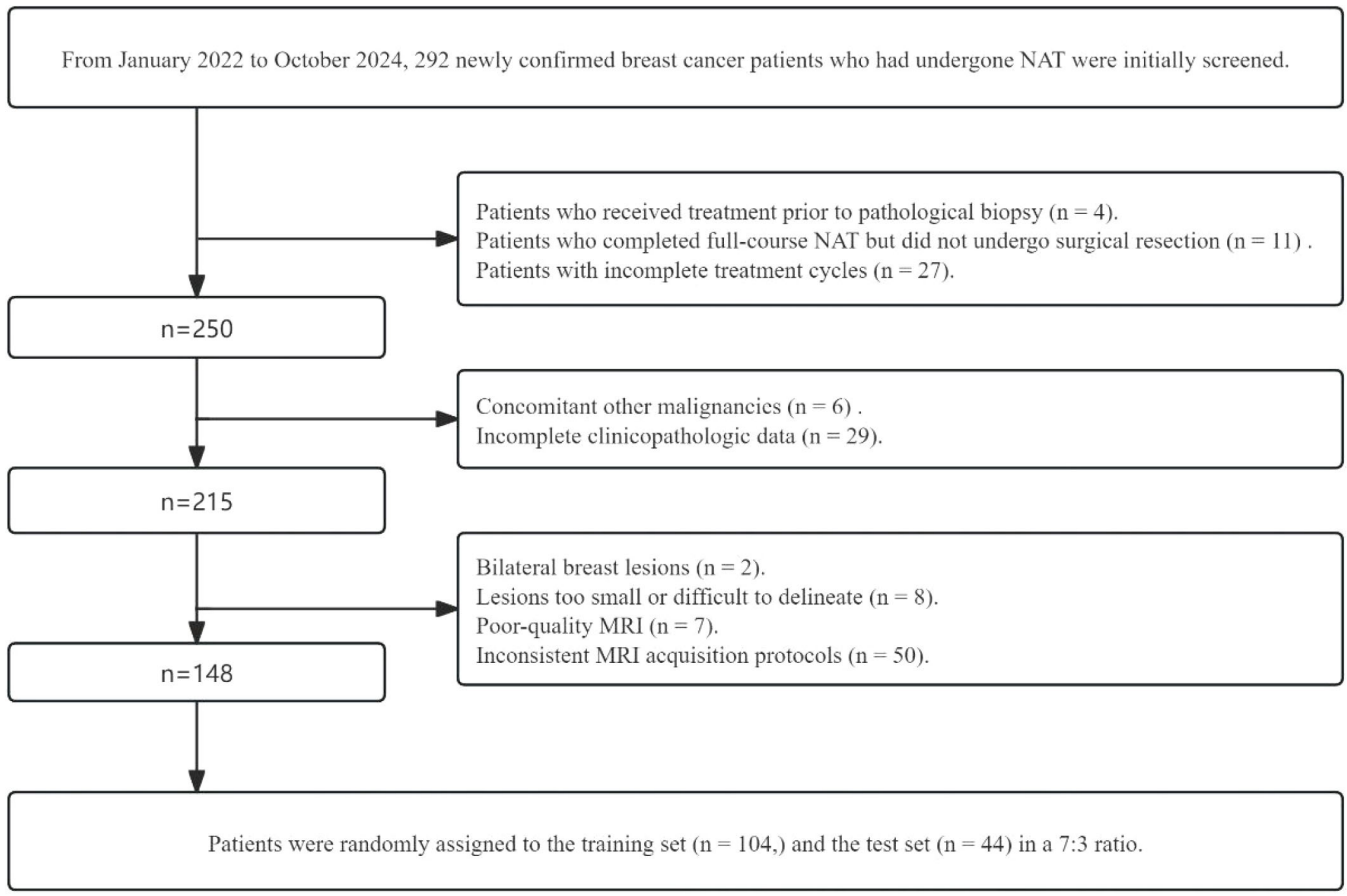
Flowchart of patient cohort and train-test split.

This ethics committee-approved retrospective study consecutively enrolled 148 breast cancer patients with pathological confirmation undergoing NAT between January 2022 and October 2024 from the Center 1. Inclusion criteria: (i) Biopsy-confirmed diagnosis with baseline Ki-67; (ii) Post-NAT surgical resection with Ki-67 reassessment; (iii) Complete pre-NAT MRI scans; (iv) Complete imaging and clinicopathologic data. Exclusion criteria: (i)No history of other malignancy; (ii) Non-surgical management post-NAT; (iii) Incomplete clinical/imaging records; (iv) Incomplete NAT regimens or concurrent immunotherapy. **Figure 1** details the patient selection flowchart.

### 2.2 Clinicoradiological data acquisition

Clinicopathological data included demographics (BMI, menopausal status), serum biomarkers (CEA, CA153, CA125), pathological features (histological type/grade, ER/PR/HER2 status, molecular subtype), and pre-/post-NAT Ki-67 (18); imaging assessments covered tumor morphology and enhancement kinetics. Breast cancer is molecularly classified into four subtypes: Luminal A, Luminal B, HER2-enriched, and Triple-negative (19, 20).Ki-67 downgrading is defined as a reduction from high expression(>20%)pre-treatment to low expression(≤20%) post-treatment (20, 21), cases not meeting this criterion are considered non-downgrading.

### 2.3 MRI protocol

All patients underwent MRI examinations on a 3.0T scanner (GE Signa Architect; GE Healthcare, Milwaukee, USA) using an 8-channel dedicated breast phased-array coil. Patients were positioned prone with both breasts naturally suspended within the coil. Axial fat-suppressed DCE-MRI scanning parameters were as follows: TR/TE 4.3/2.1 ms, FOV 320 mm × 320 mm, matrix 320 × 320, slice thickness/gap 1.4/1.4 mm, 8 phases. One pre-contrast phase was acquired as a mask, followed by intravenous bolus injection of Gadobutrol (0.2 mL/kg, 2.0 mL/s). The first post-contrast phase was initiated approximately 60 seconds after injection, followed by six consecutive acquisitions. Each phase took 60 seconds (total scan time: 8 min).

### 2.4 Image segmentation and radiomics feature extraction

**Figure 2:**
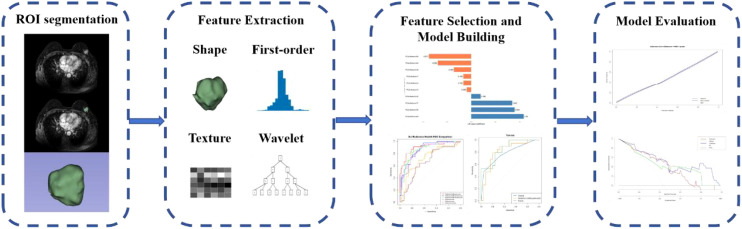
Radiomics workflow diagram: ROI segmentation delineation, feature extraction, feature selection, model building, and model evaluation.

All patients’ DCE-MRI images were acquired via the Picture Archiving and Communication System (PACS). Three contrast-enhanced phases were selected: Phase 2 (early), peak enhancement phase (time-intensity curve analysis), and Phase 8 (terminal). DICOM images were imported into 3D Slicer software (v5.2.1; Boston, MA, USA) and resampled to 1×1×1 mm³ voxel resolution. Two radiologists, each with over 3 years of experience in breast MRI diagnosis, performed slice-by-slice delineation of breast lesions using a combination of manual and semi-automatic methods. In cases of disagreement, a senior radiologist with 10 years of diagnostic experience reviewed the delineated ROIs as the final arbiter (**Figure 2**). We randomly selected 30 patients’ MRI images and repeated the tumor segmentation process to calculate the intra-class correlation coefficient (ICC) to evaluate the stability of the extracted radiomic features.

Delineated volumes of interest (VOIs) were performed feature extraction within 3D Slicer software. 851 radiomics features (shape, first-order, wavelet, texture features) were extracted per phases (early, peak, terminal) from VOIs segmented. Delta features were computed as follows:

Radiomics-Delta_terminal-peak_=Terminal phase features – Peak phase features. Radiomics-Delta_terminal-early_ = Terminal phase features – Early phase features. Radiomics-Delta_peak-early_ = Peak phase features – Early phase features.

### 2.5 Radiomics model development

After standardization of features using Z-score normalization, feature selection was performed using FeAture Explorer Software(FAE), a toolkit developed based on Python (version 3.7.6) (22). This study strictly adhered to standard machine learning protocols for model development and validation. The primary dataset was randomly divided into a training set (70%, 104 patients) and a test set (30%, 44 patients) at a 7:3 ratio. The test set was completely isolated from the outset and did not participate in any form in the subsequent model development or selection. Model selection was performed within the training set using 5-fold cross-validation: the training set was further partitioned into five folds, with four folds used as a sub-training set and one fold as a validation set in each iteration. In each fold, all steps, including feature selection and dimensionality reduction, were fitted based solely on the current sub-training set and then applied to the corresponding validation set. The FAE software was used to automate this process, comparing multiple feature selection strategies and classifier combinations, and the optimal model was determined based on the average AUC of the cross-validation. To reduce dimensionality during feature processing, principal component analysis (PCA) was first applied. This classical technique uses a linear transformation to convert the original features into a set of uncorrelated principal components. As a result, it effectively reduces the number of variables, simplifies the data structure, and eliminates noise and redundant information, all while retaining the key information (23). On this basis, two feature selection methods—Analysis of Variance (ANOVA) and Recursive Feature Elimination (RFE)—were further combined to screen the most discriminative features. ANOVA evaluates the discriminative power of each feature by calculating its F-value with respect to the labels, ranks them by importance, and thereby selects features significantly associated with the labels. RFE, on the other hand, iteratively removes less contributive features and ultimately retains the most predictive feature subset, which helps reduce overfitting risk and enhances the model’s generalization performance. Finally, three machine learning algorithms—Support Vector Machine (SVM), Linear Discriminant Analysis (LDA), and Logistic Regression via Lasso (LR-Lasso)—were adopted to construct predictive models based on the selected features. In this study, the default parameter settings of the FAE software were adopted to avoid subjective bias introduced by manual tuning. The default parameter grid of FAE covers the common hyperparameter space of each classifier and automatically selects the optimal combination through 5-fold cross-validation, thereby minimizing the impact of researchers’ subjective intervention on model performance. Based on optimized feature subsets, six models predicting Ki-67 downgrading reduction were developed: three single-phase models (Radiomics-early, Radiomics-peak, Radiomics-terminal)and three Delta-radiomics models (Radiomics-Delta_terminal-peak_, Radiomics-Delta_terminal-early_, Radiomics-Delta_peak-early_).

### 2.6 Integrated clinical-radiomics model development

Multivariable logistic regression analyses were performed on collected clinical indicators to identify independent risk factors of Ki-67 downgrading reduction. Based on these factors, we constructed a clinical-radiomics integrated model and calculated the optimal radiomics Rad-score. A nomogram incorporating independent risk factors was developed and assessed.

### 2.7 Statistical analyses

Statistical analyses were performed using IBM SPSS Statistics 27.0, FAE (version 0.5.13), and R (version 4.5.0). Inter-group differences (training vs. test sets) were analyzed using t-tests (normal distribution), Mann-Whitney U tests (non-normal), or Chi-square/Fisher’s exact test (categorical). Two-tailed tests were employed (P<0.05). The diagnostic performance of models was evaluated using AUC, with DeLong’s test comparing AUC differences. Calibration curves and decision curve analysis (DCA) were utilized to assess the predictive accuracy and the net benefit of the different models.

## 3 Results

### 3.1 Clinical baseline characteristics of the study cohort

This study enrolled 148 patients from Center 1, who were randomly divided into a training set (n = 104) and a test set (n = 44) at a ratio of 7:3 from the Center 1. To further verify the model’s performance, we additionally included 49 patients from apartner hospital Cener 2 from November 2024 to May 2025 as an external validation. **Table 1** shows the baseline characteristics of the training set, test set, and external validation.

**Table 1:** Clinical and imaging baseline characteristics of training、test cohorts and external validation.

Baseline characteristics	Training (n=104)	Test (n=44)	*p*-value	External validation (n=49)
Age	51.61 ± 9.61	52.09 ± 9.73	0.782	50.90 ± 9.42
BMI (Kg/m^2^)	24.22 ± 2.75	24.55 ± 2.65	0.242	23.69 ± 3.00
Menstrual status, n (%)			0.992	
Pre-menopausal	45 (43.3)	19 (43.2)		27 (55.1)
Menopausal	59 (56.7)	25 (56.8)		22 (44.9)
Histologic grade, n (%)			0.238	
Grade I	5 (4.8)	1 (2.3)		1 (2.0)
Grade II	58 (55.8)	31 (70.4)		35 (71.5)
Grade III	41 (39.4)	12 (27.3)		13 (26.5)
Clinical T stage, n (%)			0.900	
T1/2	89 (85.6)	38 (86.4)		37 (75.5)
T3/4	15 (14.4)	6 (13.6)		12 (24.5)
Axillary lymph-node metastasis, n (%)			0.571	
Yes	61 (58.7)	28 (63.6)		29 (59.2)
No	43 (41.3)	16 (36.4)		20 (40.8)
HER-2, n (%)			0.310	
Negative	66 (63.5)	24 (54.5)		28 (57.1)
Positive	38 (36.5)	20 (45.5)		21 (42.9)
PR, n (%)			0.292	
Negative	50 (48.1)	17 (38.6)		19 (38.8)
Positive	54 (51.9)	27 (61.4)		30 (61.2)
ER, n (%)			0.582	
Negative	38 (36.5)	14 (31.8)		14 (28.6)
Positive	66 (63.5)	30 (68.2)		35 (71.4)
Ki-67,n (%)			0.709	
Responders	46 (44.2)	18 (40.9)		18 (36.7)
Non-responders	58 (55.8)	26 (59.1)		31 (63.3)
Molecular subtype, n (%)			0.543	
Luminal A	17 (16.3)	7 (15.9)		10 (20.4)
Luminal B	51 (49.0)	24 (54.6)		26 (53.1)
HER2-enriched	20 (19.2)	10 (22.7)		8 (16.3)
Triple-negative	16 (15.4)	3 (6.8)		5 (10.2)
CEA, n (%)			0.477	
Normal	97 (95.1)	42 (97.7)		46 (93.9)
Elevated	5 (4.9)	1 (2.3)		3 (6.1)
CA125, n (%)			0.317	
Normal	94 (90.4)	41 (95.3)		48 (98.0)
Elevated	10 (9.6)	2 (4.7)		1 (2.0)
CA153, n (%)			0.302	
Normal	94 (90.4)	39 (88.6)		44 (89.8)
Elevated	10 (9.6)	5 (11.4)		5 (10.2)
Location,n (%)			0.117	
Left	54 (51.9)	29 (65.9)		24 (49.0)
Right	50 (48.1)	15 (34.1)		25 (51.0)
Number of lesions,n (%)			0.143	
Single	91 (87.5)	42 (95.5)		42 (85.7)
Multiple	13 (12.5)	2 (4.5)		7 (14.3)
Tumor shape,n (%)			0.121	
Regular	17 (16.3)	3 (6.8)		0 (0)
Irregular	87 (83.7)	41 (93.2)		49 (100)
Tumor margin, n (%)			0.742	
Spiculated	68 (65.4)	30 (68.2)		32 (65.3)
Non-spiculated	36 (34.6)	14 (31.8)		17 (34.7)
Enhancement pattern, n (%)			0.102	
Rim	14 (13.5)	7 (15.9)		11 (22.5)
Relatively homogeneous	10 (9.6)	0 (0)		5 (10.2)
Heterogeneous	80 (79.9)	37 (84.1)		33 (67.3)
TIC curves, n (%)			0.105	
Wash-in	12 (11.6)	4 (9.1)		5 (10.2)
Plateau	43 (41.3)	11 (25.0)		18 (36.7)
Wash-out	49 (47.1)	29 (65.9)		26 (53.1)
FGT, n (%)			0.934	
a	6 (5.8)	2 (4.6)		1 (2.0)
b	22 (21.1)	11 (25.0)		13 (26.5)
c	61 (58.7)	24 (54.5)		31 (63.3)
d	15 (14.4)	7 (15.9)		4 (8.2)
BPE, n (%)			0.472	
Minimal/Mild	62 (59.6)	29 (65.9)		31 (63.3)
Moderate/Marked	42 (40.4)	15 (34.1)		18 (36.7)
Intratumoral T2 signal, n (%)			0.590	
Hyperintense	71 (68.3)	32 (72.7)		
Hypointense	33 (31.7)	12 (27.3)		18 (36.7)
Edema, n (%)			0.199	31 (63.3)
Yes	86 (82.7)	40 (90.9)		41 (83.7)
No	18 (17.3)	4 (9.1)		8 (16.3)

TIC, time-signal intensity; FGT, fibroglandular tissue; BPE, breast parenchymal enhancement; p-value>0.05 indicates no significant statistical difference between the training set and the test set.

### 3.2 Clinical-imaging model

Univariable analysis identified PR, ER, HER2 status, histologic grade, molecular subtype, lymph node metastasis, and spiculated margins as significantly associated with Ki-67 downgrading(P<0.05). Multivariable logistic regression confirmed HER2-enriched (OR:2.827; 95% CI:1.098-7.278) and higher histologic grade (OR: 7.508; 95% CI: 2.752-20.486) as independent predictors of Ki-67 downgrading (**Table 2**).

**Table 2:** Multivariable logistic regression analysis of clinical and imaging variables.

Baseline characteristics	Multivariable logistic regression analysis		
	OR	95% CI	*p*-value
HER2	2.601	(1.038-6.517)	0.041*
PR	0.358	(0.107-1.199)	0.096
ER	0.758	(0.141-4.080)	0.747
Molecular subtype	0.997	(0.366-2.718)	0.995
Histologic grade	6.260	(2.460-15.932)	<0.001*
Axillary lymph-node metastasis	0.829	(0.331-2.076)	0.688
Tumor margin	0.448	(0.173-1.162)	0.099

OR, Odds Ratio; 95% CI, 95% Confidence Interval; *indicates statistical significance.

Bolded numbers are denoted by * for p < 0.05, indicating statistical significance.

### 3.3 Radiomics models performance and comparison

We employed two feature selection methods, ANOVA and RFE, in combination with three classifiers—SVM, LDA, and LR-Lasso—to select and model single-phase and delta radiomics features. The corresponding AUC values are summarized in the table below (**Table 3**).

**Table 3:** Predictive performance of different radiomics models in training and test sets.

Radiomics		SVM		LDA		Lasso	
		Training (95% CI)	Test (95% CI)	Training (95% CI)	Test (95% CI)	Training (95% CI)	Test (95% CI)
ANOVA	Radiomics- Delta_peak-early_	0.889 (0.824-0.953)	0.813 (0.679-0.947)	0.881 (0.813-0.950)	0.813 (0.679-0.946)	0.910 (0.856-0.964)	0.796 (0.658-0.934)
	Radiomics- Delta_terminal-peak_	0.834 (0.756-0.913)	0.585 (0.412-0.759)	0.839 (0.762-0.915)	0.568 (0.394-0.743)	0.841 (0.764-0.918)	0.577 (0.403-0.751)
	Radiomics- Delta_terminal-early_	0.850 (0.776-0.923)	0.627 (0.457-0.798)	0.856 (0.784-0.929)	0.648 (0.481-0.816)	0.846 (0.772-0.920)	0.621 (0.452-0.790)
	Radiomics-Peak	0.753 (0.660-0.846)	0.585 (0.408-0.762)	0.879 (0.814-0.945)	0.610 (0.460-0.759)	0.875 (0.806-0.944)	0.562 (0.385-0.739)
	Radiomics- _terminal_	0.663 (0.556-0.769)	0.522 (0.343-0.702)	0.807 (0.723-0.891)	0.563 (0.412-0.715)	0.662 (0.556-0.769)	0.522 (0.343-0.702)
	Radiomics-early	0.793 (0.705-0.881)	0.562 (0.388-0.736)	0.826 (0.747-0.905)	0.530 (0.378-0.681)	0.866 (0.799-0.933)	0.579 (0.403-0.755)
RFE	Radiomics- Delta_peak-early_	0.904 (0.824-0.953)	0.802 (0.679-0.947)	0.908 (0.847-0.969)	0.815 (0.682-0.948)	0.905 (0.846-0.965)	0.817 (0.685-0.949)
	Radiomics- Delta_terminal-peak_	0.852 (0.777-0.928)	0.587 (0.410-0.765)	0.858 (0.785-0.931)	0.583 (0.407-0.759)	0.860 (0.787-0.933)	0.579 (0.402-0.756)
	Radiomics- Delta_terminal-early_	0.873 (0.806-0.940)	0.679 (0.519-0.839)	0.873 (0.807-0.939)	0.688 (0.529-0.848)	0.872 (0.807-0.938)	0.648 (0.484-0.812)
	Radiomics-Peak	0.873 (0.802-0.945)	0.625 (0.458-0.793)	0.713 (0.613-0.812)	0.590 (0.412-0.767)	0.841 (0.766-0.915)	0.615 (0.444-0.785)
	Radiomics- _terminal_	0.663 (0.556-0.769)	0.522 (0.343-0.702)	0.662 (0.556-0.769)	0.522 (0.343-0.702)	0.663 (0.556-0.769)	0.522 (0.343-0.702)
	Radiomics-early	0.727 (0.627-0.828)	0.547 (0.370-0.724)	0.779 (0.690-0.869)	0.443 (0.293-0.593)	0.724 (0.623-0.825)	0.537 (0.360-0.714)

95% CI: 95% Confidence Interval.

Based on the results summarized in the table above, the test set AUC values of different models for predicting Ki-67 downgrading are as follows: the Radiomics-Delta_peak-early_ model generally achieved the highest performance. Regarding feature‐model combinations, although the ANOVA + LR-Lasso combination attained the highest training set AUC with Radiomics-Delta_peak-early_ features, its test set AUC was significantly lower, indicating overfitting. The ANOVA + SVM combination performed poorly in predicting both Radiomics-Delta_terminal-early_ and Radiomics-early, as reflected by low AUC values. The RFE + LDA combination yielded the lowest test set AUC on Radiomics-early, while the ANOVA + LDA combination achieved a lower training set AUC with Radiomics-Delta_peak-early_ features compared to the RFE + SVM and RFE + LR-Lasso combinations. Overall, considering both training and test performance, the RFE + SVM and RFE + LR-Lasso combinations demonstrated the best generalizability and stability across tasks. Ultimately, we selected the optimal Radiomics-Delta_peak-early_ model, which demonstrated the highest performance in both training and test sets using the RFE + LR-Lasso configuration, as our final reporting model.

**Figure 3:**
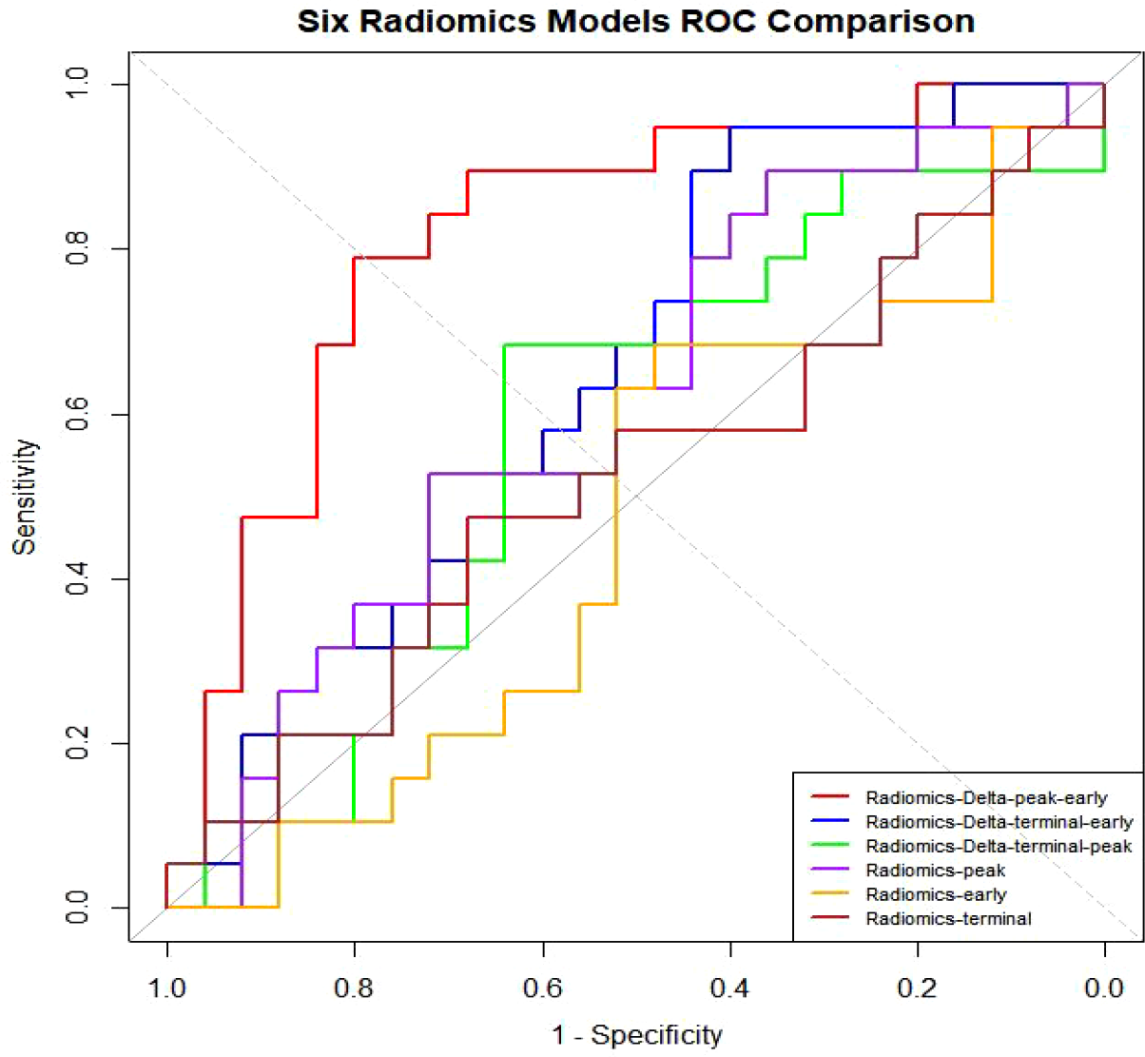
ROC curves for six radiomics models, demonstrating that the Radiomics-Delta_peak-earlty_ model achieved the highest AUC.

**Figure 4:**
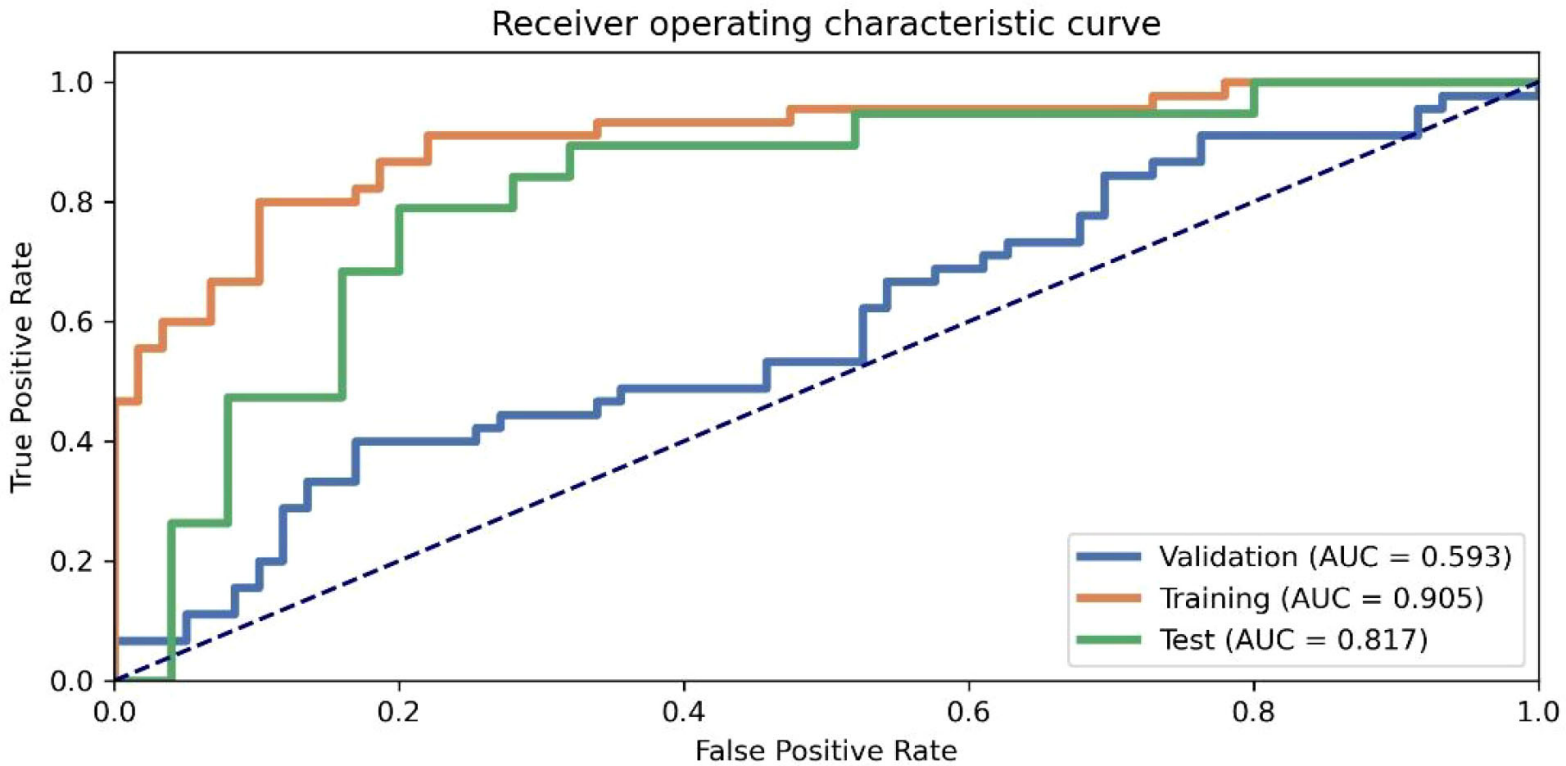
Model performance generated by RFE and ROC curves of the model across different datasets.

Based on the RFE + LR-Lasso feature selection strategy, we performed feature screening and model comparison within the training set using 5-fold cross-validation. A total of four PCA-derived features were selected from early-phase images, nine from peak-phase images, and one from late-phase images. Furthermore, ten features were obtained from the Delta (peak-early) comparison, eight from the Delta (terminal-early), and seven from the Delta (terminal-peak). The DeLong test revealed that the AUC of the proposed model was significantly superior to those of other candidate models (P < 0.05), and it achieved the highest sensitivity and specificity in the test set (**Figure 3**). Ultimately, ten principal components derived from the Delta (peak-early) features were selected to construct the optimal predictive model using the LR-Lasso classifier. This model yielded AUCs of 0.905 (95% CI: 0.846–0.965) in the training set and 0.817 (95% CI: 0.685–0.949) in the test set, with an average cross-validation AUC of 0.596 (**Figure 4**).

### 3.4 Clinical-radiomics integrated model and predictive nomogram

**Figure 5:**
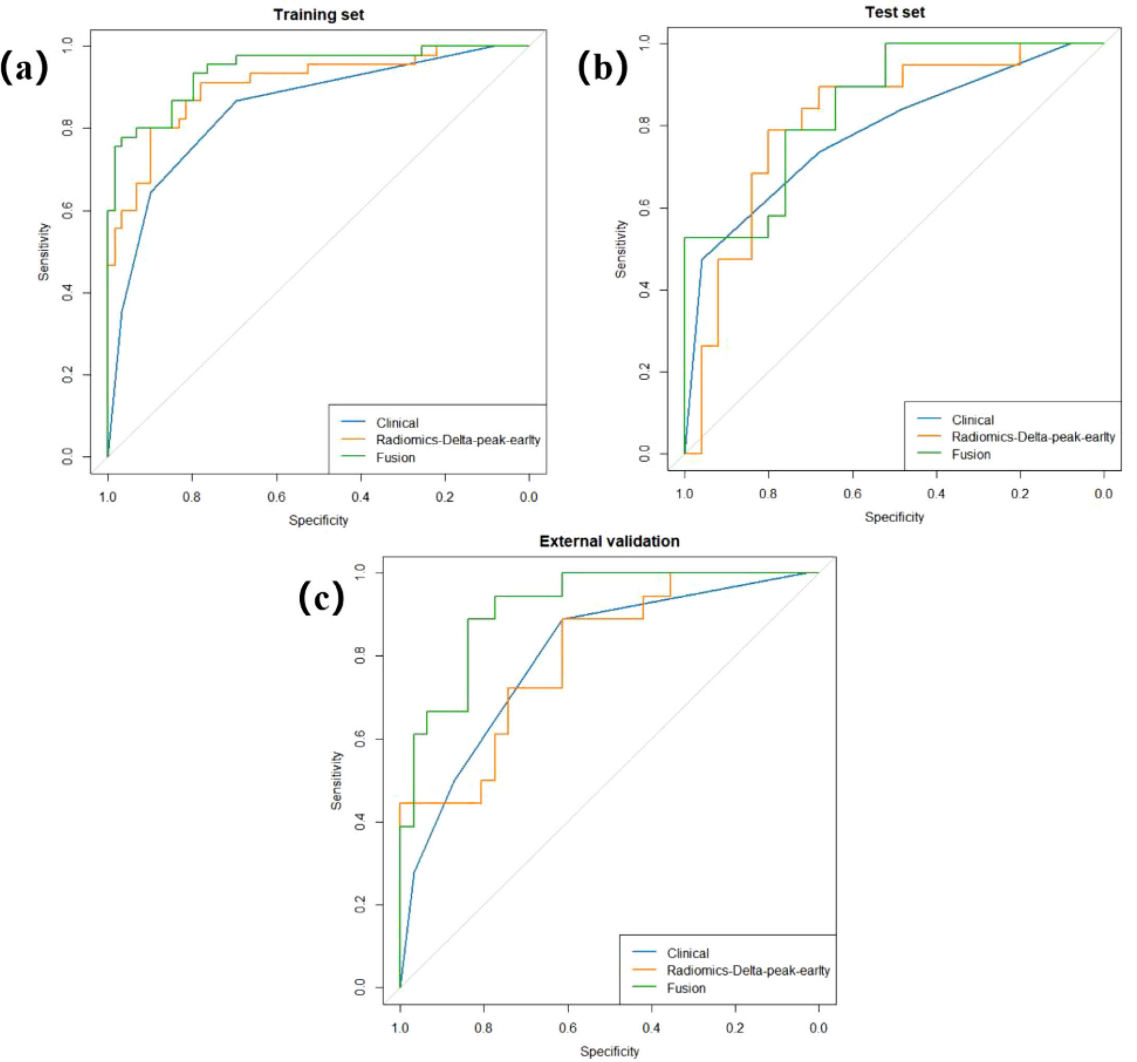
Results of ROC analysis for Clinical, Radiomics-Delta, and Radiomics-Clinical models in the training **(a)**, test **(b)**, and external validation **(c)** sets. The integrated model achieved the highest area under the curve in all three sets.

The optimal radiomics model (Radiomics-Delta_peak-early_) was integrated with independent clinical predictors (HER2 status, histologic grade) to construct a combined model. The integrated model achieved the highest AUC (p < 0.05), with 0.943 (95% CI: 899-0.987) in training set (**Figure 5a**, **Table 4**) and 0.851 (95% CI: 0.740-0.960) in test set (**Figure 5b**, **Table 4**).

**Table 4:** Comparative performance analysis of clinical, Delta radiomics, and clinical-Delta radiomics integrated models.

Models		Clinical	Radiomics-Delta_peak-earlty_	Fusion
Training	AUC	0.848	0.905	0.943
	Sen	0.867	0.800	0.800
	Spe	0.695	0.898	0.949
	95% CI	0.775-0.921	0.846–0.965	0.899–0.987
Test	AUC	0.785	0.817	0.851
	Sen	0.474	0.790	0.789
	Spe	0.960	0.800	0.760
	95% CI	0.649-0.922	0.685–0.949	0.740–0.961
External validation	AUC	0.801	0.803	0.919
	Sen	0.889	0.889	0.889
	Spe	0.613	0.613	0.839
	95% CI	0.680–0.924	0.678–0.928	0.846–0.992

Sen, Sensitivity; Spe, Specificity.

Radscore is a composite numerical index derived by employing specific mathematical models or algorithms to filter, weight, and linearly combine multiple high-throughput extracted radiomic features, ultimately condensed through dimensionality reduction. The calculation formula is as follows:


$$R{\text{adscore}}_{i}\;=\;\beta_{0}\;+\;\sum_{\text{j}=1}^{\text{p}}\beta_{j}\;\cdot \;X_{\text{ij}}$$

(i represents the i-th sample; p denotes the number of principal components retained (after PCA+RFE); β_0_ represents the model intercept; β_j_ denotes the weight of the j-th retained feature; X_ij_ represents the value of the i-th sample on the j-th retained feature).

**Figure 6:**
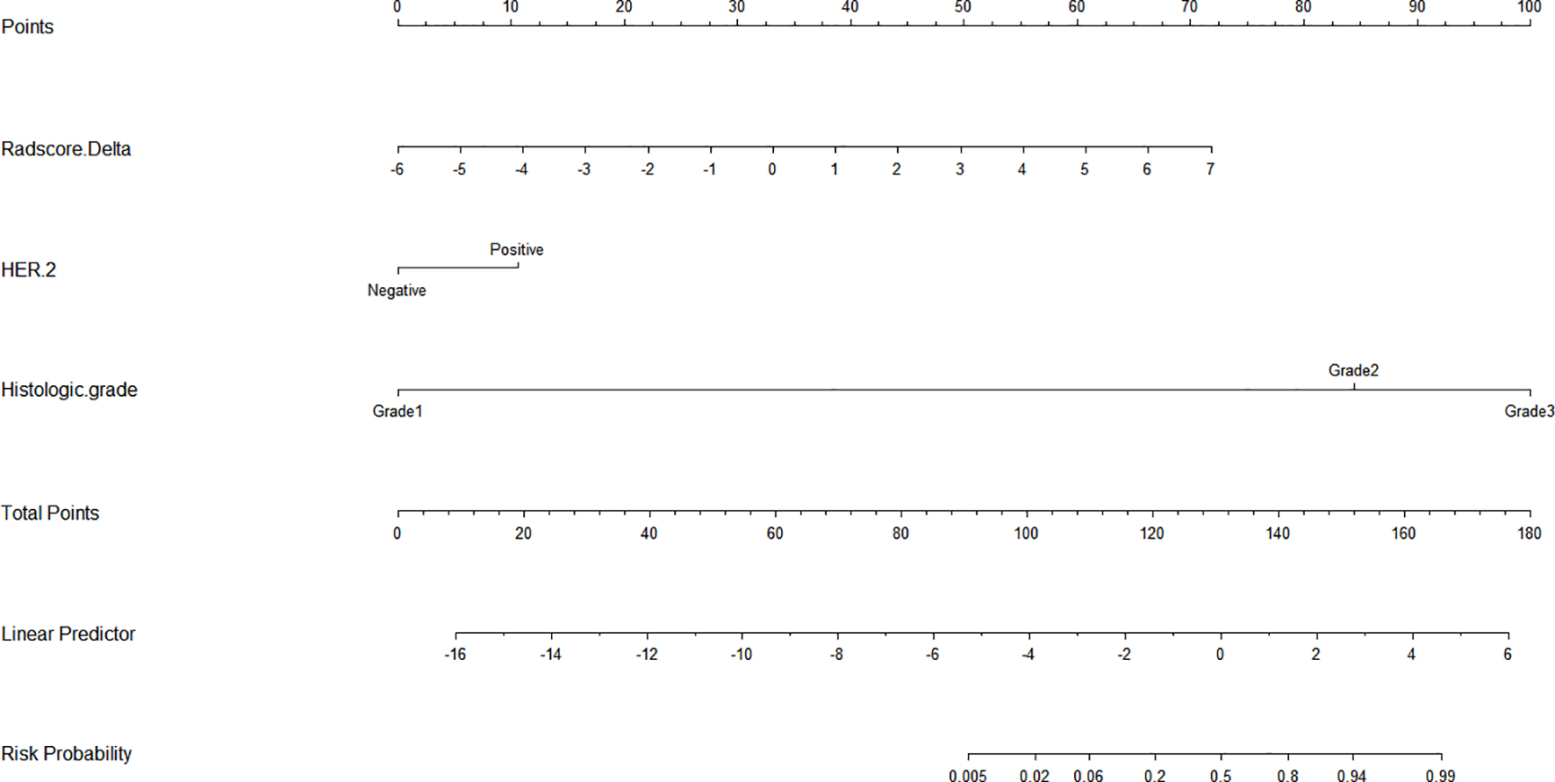
The nomogram based on the integrated model for assessing the risk of Ki-67 reduction.

**Figure 7:**
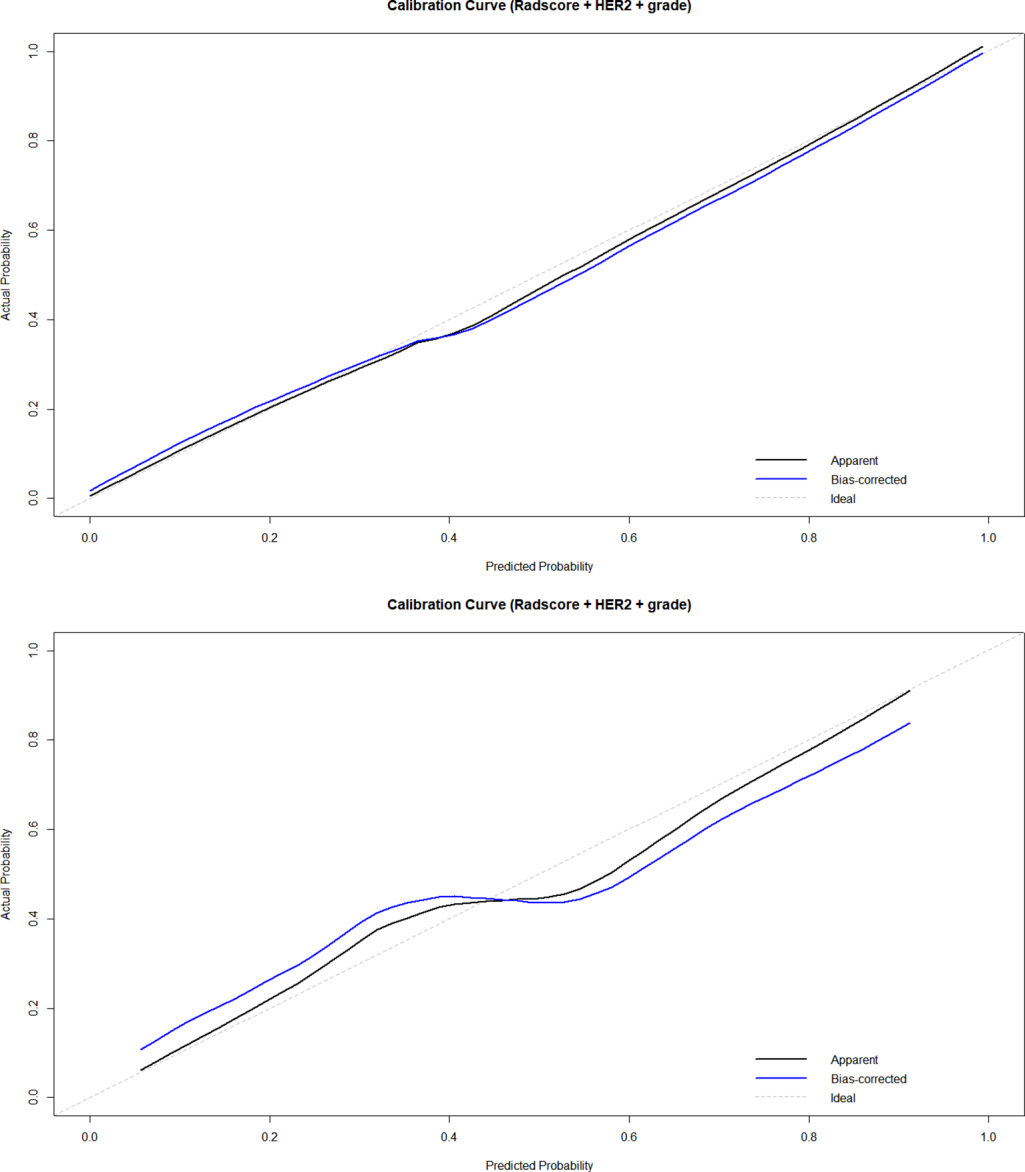
The calibration curve of the nomogram.

**Figure 8:**
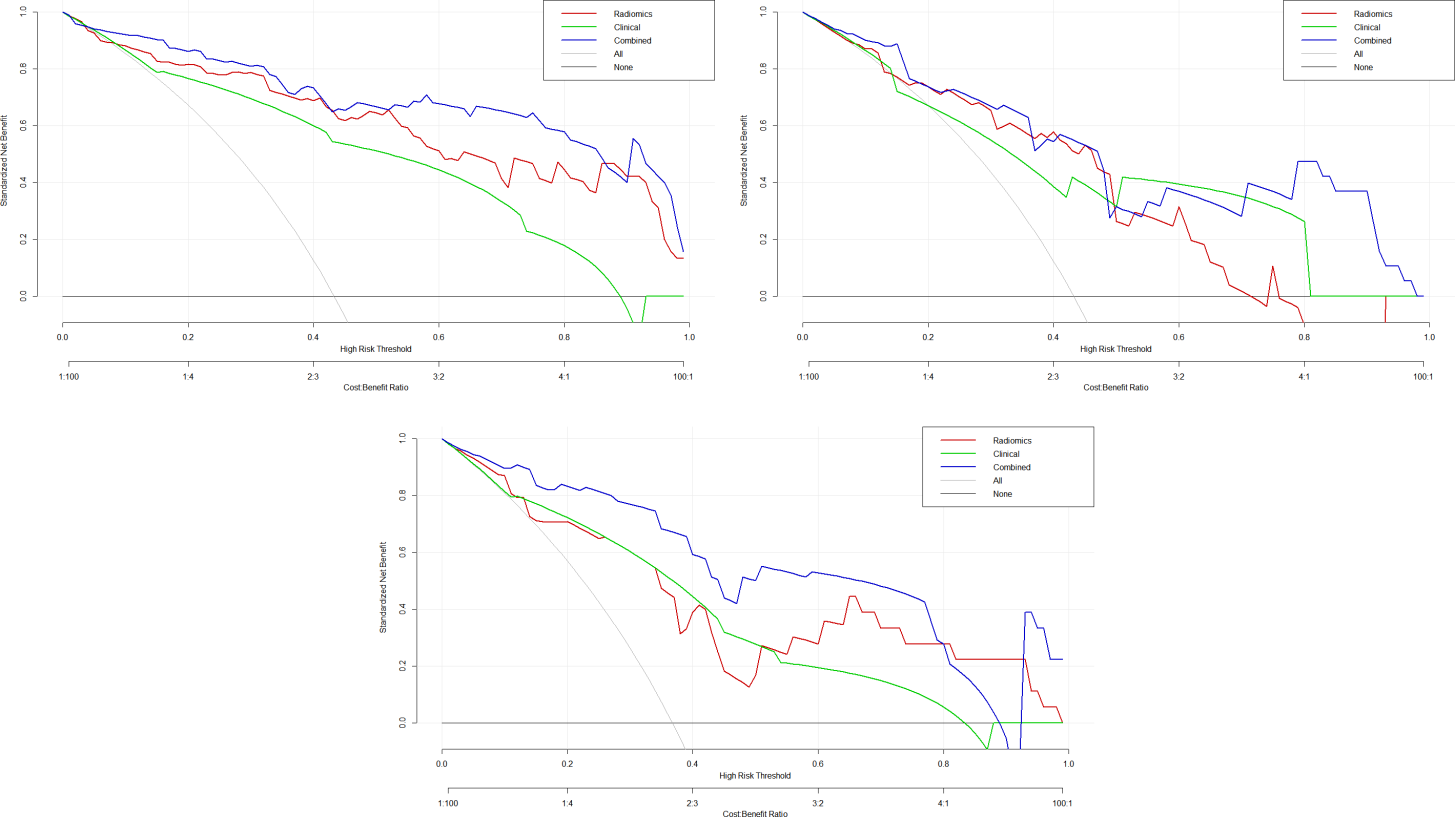
The decision curves for Radiomics-Delta, clinical, and integrated models.

Subsequently, a nomogram incorporating the Radscore, HER2 status, and histological grade was developed (**Figure 6**). The nomogram serves as a graphical computing tool based on a regression model, designed to amalgamate complex radiomic models with traditional clinical/pathological parameters. It offers a quantitative and personalized risk prediction for individual patients in a visual format. To validate this tool, calibration analysis was performed. The calibration curves, which are graphical tools for assessing the agreement between a model’s predicted probabilities and the actual observed outcomes, indicated a high level of consistency between the predicted and observed frequencies, thereby further verifying the nomogram’s reliability (**Figure 7**). Additionally, decision curve analysis (DCA)—a graphical analytical method for evaluating the practical value of clinical prediction models—was conducted. The DCA demonstrated that across a threshold probability range of >20%, the net benefit of our model was significantly superior to a “no-intervention” strategy. Moreover, within the 60-80% threshold probability range, the net benefit of the nomogram was higher than that of both the radiomics model and the clinical-imaging feature model (**Figure 8**).

To further evaluate the stability of the model, we included breast cancer patients from Center 2 as an external validation cohort. Data processing followed the same procedure as previously described: dimensionality reduction and feature selection were performed using PCA combined with RFE, and the LR-Lasso classifier was applied to the external dataset for testing. In the external validation, the Radiomics-Delta_peak-early_ model achieved an AUC of 0.803 (95% CI: 0.678–0.928), with a sensitivity of 0.889 and a specificity of 0.613. The clinical model yielded an AUC of 0.801 (95% CI: 0.680–0.924), while the combined model integrating Radiomics-Deltapeak-early features attained an AUC of 0.919 (95% CI: 0.846–0.992), outperforming both the clinical-only and radiomics-only models (**Figure 5c**, **Table 4**).

## 4 Discussion

This study establishes the superiority of Radiomics-Delta, particularly the Radiomics-Delta_peak-early_ model, outperforming single-phase radiomics in predicting Ki-67 downgrading post-NAT in breast cancer. Building upon these findings, we developed an fusion model incorporating the optimal radiomics model (peak-early) with clinically significant predictors (HER2 status and histological grade). This fusion model demonstrated superior diagnostic efficacy, exhibiting potential discrimination, calibration, and clinical utility.Preliminary temporal external validation has confirmed the model’s potential generalization capability and clinical applicability. Its promising performance in predicting dynamic changes in Ki-67 following neoadjuvant therapy lays the groundwork for developing it into a reliable tool for precision medicine decision-making.

Previous studies have established the feasibility of MRI-based radiomics models for predicting Ki-67 expression status (24). However, these models relied exclusively on single-phase features and demonstrated limited diagnostic performance in clinical validation. Lu et al. (25) developed an XGBoost model combining clinical factors with ultrasound delta-radiomics, identifying lymph node metastasis, tumor volume, elasticity score, and Delta-rad-score as independent predictors using Ki-67 ≥15% as threshold. Their approach quantified tumor changes before and after NAT, captured therapy-induced biological shifts, and significantly improved Ki-67 prediction. Zheng et al. (26) compared DCE-MRI radiomics models using pre-contrast (A0), first-phase enhancement (A1), and subtraction sequences (Delta), demonstrating the superior performance of Radiomics-Delta in assessing lymphovascular invasion. This suggests delta features can dynamically track angiogenesis and hemodynamic heterogeneity to reflect tumor aggressiveness. While prior research predominantly focused on baseline Ki-67 status in breast cancer (27), few investigations have addressed radiomics prediction of dynamic Ki-67 changes. Ju et al. (10) established the optimal cutoff for NAT-induced Ki-67 index changes and revealed its prognostic value for breast cancer outcomes. Building on this, we hypothesize that DCE-MRI radiomics can predict dynamic Ki-67 changes during NAT, with delta-radiomics outperforming single-phase features in characterizing Ki-67 trajectories, thus improving response prediction and risk stratification. Analysis of clinical and imaging features identified HER2 status and histologic grade as independent predictors of Ki-67 reduction. Previous studies show ER+, HER2-low tumors are less NAT-sensitive, while HER2+ status and higher histologic grade correlate with higher pCR rates (28, 29). Our findings align with evidence linking HER2+ status and higher histologic grade to Ki-67 reduction. ER+ did not reach independent significance, possibly due to cohort heterogeneity. Thus, HER2 status and grade were included in the final model, which—combined with delta-radiomics—showed strong performance in predicting Ki-67 reduction.

Delta-radiomics features show time-dependent predictive performance linked to tumor enhancement kinetics. DCE-MRI captures microcirculatory changes via post-contrast tissue enhancement. Early-phase images offer high lesion-background contrast, while peak phase shows maximum enhancement. Rapidly proliferating tumors (often high Ki-67) typically have rich vascularity and high perfusion, resulting in “fast wash-in/wash-out” kinetics—rapid signal rise to peak followed by quick decline or plateau (washout/plateau pattern). Thus, hemodynamic changes between peak and early phases are most prominent, marked by large intensity increase and steep slope (30, 31). Therefore, delta-radiomics features from peak-early phases best capture these significant hemodynamic shifts, explaining their superior prediction. In contrast, plateau- or persistent-type tumors show slow enhancement with delayed peaks near the delayed phase, causing minimal change between delayed-peak phases and weaker predictive power. Thus, the peak-early delta-radiomics model achieved optimal performance. To improve Ki-67 prediction, the model was combined with key clinical features (HER2 status and histological grade) identified by multivariate logistic regression. This integrated model significantly enhanced accuracy in predicting post-neoadjuvant Ki-67 changes, achieving the highest diagnostic performance in the study. Calibration analysis confirmed its accurate risk estimation, increasing confidence in clinical applicability and supporting personalized treatment. DCA further validated its utility in individualized decision-making and patient management.

This study has several limitations: (i) Although we included patient cohorts from different time periods for temporal external validation, all data originated from a unified imaging platform and protocol. Therefore, the model’s robustness could not be evaluated across different MRI scanners, imaging parameters, or heterogeneous patient populations. These factors inevitably limit the current generalizability and clinical translation potential of our conclusions, and future multicenter prospective studies with larger sample sizes are required for further validation; (ii) Additionally, this study did not perform stratified analyses based on the four molecular subtypes of breast cancer. Subsequent research should further investigate the changes in Ki-67 expression following neoadjuvant therapy across different molecular subtypes; (iii) The link between the change of Ki-67 and long-term outcomes was not assessed, requiring prognostic evaluation in follow-up studies; (iv) In terms of methodology, the ROI delineation process presents several noteworthy limitations: the current reliance on semi-automatic manual segmentation makes it difficult to avoid inter-observer variability, resulting in inconsistent ROI definitions and challenging result reproducibility. Moving forward, future research should prioritize exploring the potential of deep learning techniques for automated lesion segmentation to establish a more objective and reproducible standardized workflow.

## 5 Conclusions

The Radiomics-Delta model demonstrated superior performance compared to single-phase radiomics. By integrating clinical and radiomic features with dynamic Radiomics-Delta signatures, the hybrid model significantly improved the prediction of Ki-67 changes following NAT in breast cancer.

## Data Availability

The raw data supporting the conclusions of this article will be made available by the authors, without undue reservation.
